# A Microstrip Transmission Line Biosensor to Measure the Interaction between Microliter Aqueous Solutions and 1.0–17.0 GHz Radio Frequencies

**DOI:** 10.3390/s23115193

**Published:** 2023-05-30

**Authors:** Mary Godfrey, Daniel Ewert, Ryan Striker, Benjamin Braaten

**Affiliations:** 1 College of Engineering, North Dakota State University, Fargo, ND 58102, USA; benjamin.braaten@ndsu.edu; 2 College of Engineering and Mines, University of North Dakota, Grand Forks, ND 58202, USA; dan.ewert@und.edu (D.E.); ryan.striker@und.edu (R.S.)

**Keywords:** RF biosensor, microstrip transmission line sensor, microliter sensor, biosensing

## Abstract

Radio frequency (RF) biosensors are an expanding field of interest because of the ability to design noninvasive, label-free, low-production-cost sensing devices. Previous works identified the need for smaller experimental devices, requiring nanoliter to milliliter sampling volumes and increased capability of repeatable and sensitive measurement capability. The following work aims to verify a millimeter-sized, microstrip transmission line biosensor design with a microliter well operating on a broadband radio frequency range of 1.0–17.0 GHz. Three successive experiments were performed to provide evidence for (1) repeatability of measurements after loading/unloading the well, (2) sensitivity of measurement sets, and (3) methodology verification. Materials under test (MUTs) loaded into the well included deionized water, Tris-EDTA buffer, and lambda DNA. S-parameters were measured to determine interaction levels between the radio frequencies and MUTs during the broadband sweep. MUTs increasing in concentration were repeatably detected and demonstrated high measurement sensitivity, with the highest error value observed being 0.36%. Comparing Tris-EDTA buffer versus lambda DNA suspended in Tris-EDTA buffer suggests that introducing lambda DNA into the Tris-EDTA buffer repeatably alters S-parameters. The innovative aspect of this biosensor is that it can measure interactions of electromagnetic energy and MUTs in microliter quantities with high repeatability and sensitivity.

## 1. Introduction

Biosensors are widely used detection devices that convert a biological response into a measurable signal(s) [1,2,3]. Biosensors are frequently used to characterize a liquid or semi-solid material’s response to electromagnetic (EM) wave exposure [1,2,3,4,5,6] operating throughout the entire EM spectrum. The field of biosensing is massive, and there are many subgroups of biosensors based on classification type (e.g., label-free vs. labeled, in vivo vs. ex vivo, direct vs. indirect sensing, etc.), operating frequency (e.g., radio, microwave, millimeter, terahertz, optical), and detection methodology (fluorescence, optics, surface plasmon resonance, absorbance, interferometry, permittivity, scattering (S) parameters (phase/magnitude/resonance frequency), etc.), which are thoroughly discussed in recent reviews [1,7,8,9,10]. These extensive review papers have comprehensive summary tables that contain a wealth of detailed biosensor information. Biosensors are designed to operate within the ionizing or non-ionizing range and oftentimes at an operation point equal to the resonant frequency of a material under test (MUT) to invoke a specific or desired biological reaction or effect. Meanwhile, other biosensors are designed for targeted operation points. Many fall within the Industrial, Scientific, and Medical (ISM) frequency bands, just as popular handheld electronics do, e.g., cellular phones, smart watches, tablets, and other wireless communication devices.

Many dielectric characterization biosensors are designed and prototyped for operating at a single resonant frequency determined by the electronic circuitry’s size/shape/configuration, most commonly featuring a patch resonator [5,11,12,13] or ring resonators, e.g., split ring resonator (SRR) or complementary split ring resonators (CSRR) [3,4,14,15,16,17,18,19,20,21]. SRRs and CSRRs create an ideal circular loading area for MUTs, such as an aqueous solution.

Narrowband devices are also popular, which may include microfluidic channels [4,22,23,24,25,26,27,28,29] and/or a variety of fabricated geometric surface structures. Focusing on a specific resonant frequency or frequencies increases the sensitivity of measurements of any MUT. However, using a patch, SRR, CSRR, or microfluidic-based biosensors constrain testing to a single or narrowband of frequencies [19], which may unintentionally exclude information about interactions/effects of exposure of MUTs with/to EM waves.

The planar, microstrip transmission line radio frequency (RF) operating biosensors [23,26,30,31,32] are favored for decreasing production cost, MUT volume requirements, and compactness, meanwhile increasing design flexibility for broadband operating ranges and non-invasive, label-free, real-time testing. A planar, microstrip transmission line RF biosensor with dielectric spectroscopic functionality offers increased insight into topics of interest, including: (1) whether exposing MUT to EM energy has a subcellular effect on the interaction of forces between the transmitted EM energy and the affected MUT or (2) whether the presence/absence of a MUT within electric (E-field) or magnetic (H-field) field lines affects the voltages/currents in each respective field, indicating the MUT altering or being altered by EM energy exposure. 

Improving and expanding upon current RF biosensor designs will help meet commercial, personal-use, and laboratory biosensing needs. The following paper describes, in Section 2, the experimental verification of a novel broadband, planar, microstrip transmission line RF biosensor, which operates between frequencies 1.0–17.0 GHz, and how it was previously designed and simulated for proof of concept. The design includes an etched well adjacent to a copper transmission line for holding MUTs, located inside the E-field and H-field lines of the transmitting transmission line. Scattering (S) parameters during EM wave transmission will measure S_21_ values to establish interaction levels between the EM energy and MUT held within the well. Section 3 of the paper discusses the parametric analysis performed on the experimental results and displays the proposed biosensor’s ability to distinguish varying concentrations of MUTs. Afterward, Section 4 will discuss how experimental data could indicate interaction levels between the MUT and frequencies of interest for future broadband electrical impedance biosensor designs. 

## 2. Materials and Methods

### 2.1. Biosensor Design and Fabrication

**Figure 1:**
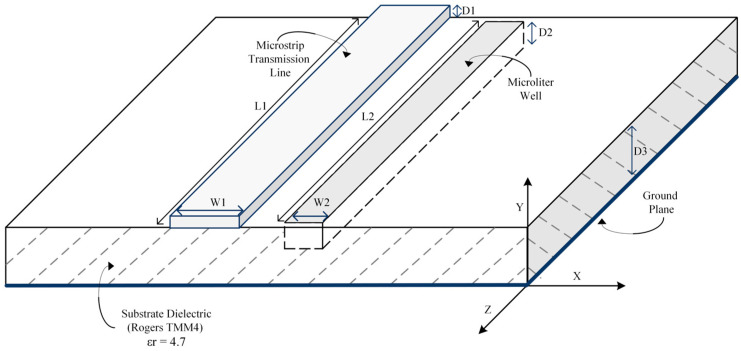
Shown is a 3D rendering of the proposed RF biosensor. A microliter well is located directly adjacent to the microstrip transmission line, which is seen milled into the substrate and will be used to hold aqueous solutions under test.

**Figure 2:**
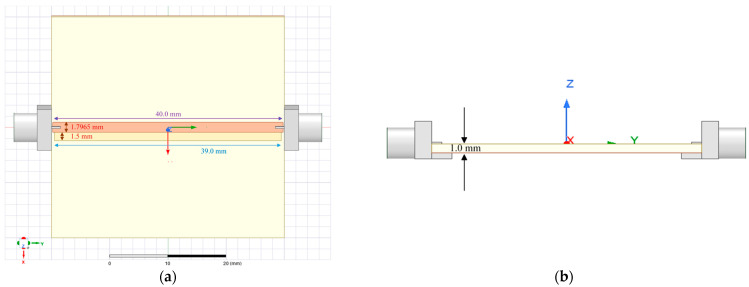
(**a**) A 3D simulation was created in HFSS of the top view of the proposed microstrip transmission line RF biosensor. Design features include using Rogers TMM4 material, a transmission line made of copper, and the adjacent microstrip well (shown in light yellow color) for loading/unloading of MUTs in simulations. (**b**) The lateral view of the biosensor, including the two SMA connectors modeled on each end for coaxial connection to a Vector Network Analyzer.

In our previous work [33], in which we used Advanced Design System (ADS) 2020 and Ansys Electronics Desktop 2021 R1 HFSS, 2D and 3D simulation software releases respectively, a proof of concept and design of a broadband, microstrip transmission line RF biosensor measuring dielectric changes by collecting S-parameters was proposed and shown in Figure 1. Although the design process and simulation steps are explained in detail in our previous work, a brief overview will be discussed here for clarity. First, 2D ADS simulations were used to determine a viable geometry for a 50-ohm termination load, with resulting dimensions shown in Table 1 and adapted from [33]. After simulation in ADS, a 3D model was realized in HFSS—shown in Figure 2—that measured S-parameters S_11_ and S_21_ through a frequency sweep of 1.0–17.0 GHz.

**Figure 3:**
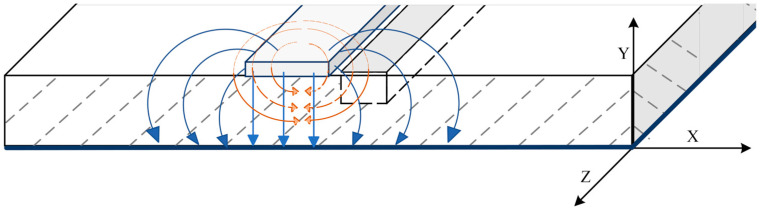
The E- and H-field radiation pattern lines are shown for a microstrip transmission line. The electric field lines (solid blue lines) radiate perpendicular to the transmission line in a clockwise and counterclockwise circular pattern. Meanwhile, the magnetic field lines (dashed orange lines) radiate parallel to the transmission line.

According to the microstrip transmission line theory, during EM wave excitation, a microstrip transmission line antenna propagates both E- and H-fields down the transmission line, according to Balanis [36]. The radiation patterns show that E-field lines radiate perpendicularly, while H-fields radiate parallel to the transmission line shown in Figure 3. The closer to the center of the microstrip transmission line, the stronger the E- and H-field lines. The authors assert that the proposed RF biosensor design’s microliter well should be fully contained within the E- and H-field lines to maximize effectiveness in detecting internal polarization shifts or voltage variations due to a MUT being exposed to EM energy. Also, the microliter well should run the entire length of the transmission line to increase the amount of interaction time spent between the MUT and the E- and H-fields. 

**Figure 4:**
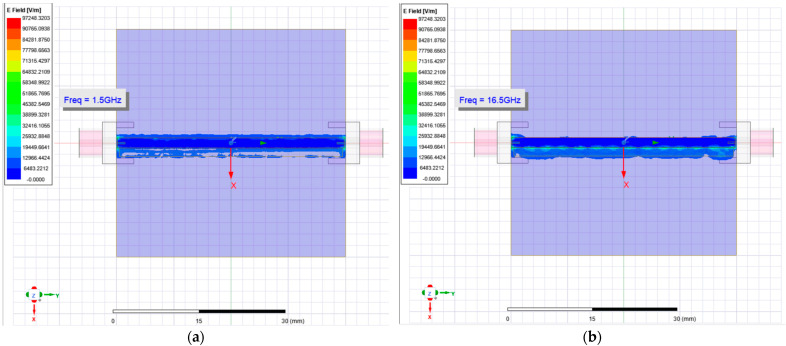
(**a**) An HFSS animation of the simulated E-field line’s strength set at 1.5 GHz during a frequency sweep from 1.0 GHz–17.0 GHz. The edges of the E-field lines pass through the entirety of the microliter well. (**b**) The simulated E-field line’s strength at a frequency point of 16.625 GHz. At higher transmitted EM wave frequency, an increase in E-field line strength is seen as the field lines continually pass through the microliter well.

Therefore, with the microliter well located on the top plane of the RF biosensor—as close to the edge of the microstrip transmission line and edge of the biosensor as allowed yet accounting for milling machine resolution and repeatability—it was incorporated into 3D Ansys HFSS simulations. E- and H-field lines were simulated for the entirety of the 1.0–17.0 GHz frequency sweep, and the distance the field lines radiated outward was determined and shown in Figure 4.

Meanwhile, Ansys HFSS simulations acted as a secondary verification to ADS for (1) a 50-ohm microstrip transmission line, (2) the addition of a 15 µL well directly adjacent to the transmission line, (3) the addition of terminal SMAs, and (4) filling the 15 µL rectangular well with MUTs, e.g., air and deionized water.

With ADS and HFSS simulations complete, Gerber files were extracted and milled using an LPKF milling machine with milling errors within ±0.3 µm mechanical resolution (X/Y) and ±1 µm repeatability [35]. Rogers TMM4, a ceramic substrate clad in copper of 17.5 µm thickness and with a dielectric constant value of 4.7 (noted in [36]), was the material used to mill the RF biosensor prototype. After the initial milling of the microstrip transmission line, a rectangular well with dimensions 39.0 mm × 0.25 mm × 1.5 mm (length x height x width) and holding an approximate volume of 15 µL was machined adjacent to the copper transmission line.

**Figure 5:**
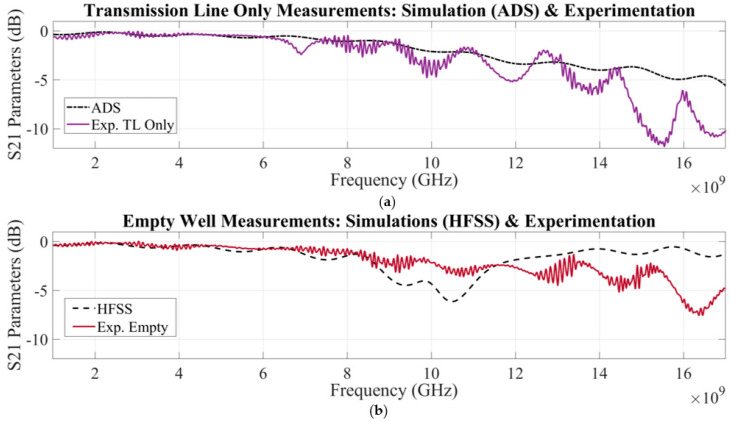
(**a**) A comparison of S_21_ parameters of the 50 Ω microstrip transmission line simulated in ADS and the experimentally tested 50 Ω microstrip transmission line prototype with exact specifications (described in Table 1) minus the milled microliter well. Note: Any effects due to SMA connectors were not accounted for in the ADS model. (**b**) After finding general S-parameter agreement in part (**a**), the prototype’s well was included. The experimental sweep protocol was reperformed with an empty well and compared to HFSS simulation results.

After fabrication and addition of SMA connectors, the RF biosensor was experimentally tested with test cases of (1) a non-loaded well and (2) a well loaded with deionized water at room temperature, and, after comparisons with ADS and HFSS, results were obtained. This study concluded that the experimental measurements were of a similar trend as the ADS and HFSS simulations, which demonstrated that the RF biosensor design could detect S-parameter changes based on a MUT being loaded into the well (shown in Figure 5).

Also, during this preliminary testing phase, a 2-channel K-type thermocouple was attached to the top and ground planes of the RF biosensor to determine if heating of the TMM4 material was taking place during experimentation. It was found that temperature values fluctuated by an average of ±0.26 °C over an experimentation time of four minutes. Therefore, it was concluded that heating of TMM4 material was not a concern and did not contribute to sample evaporation since the complete experimental sets (40–200 sweeps total with a sweep time of 65.27 ms) were collected within 2–13 s total. Finally, to test whether the RF biosensor would absorb any of the MUT during testing, the biosensor was measured (1) dry and then (2) after complete submersion in ultrapure water for 2 h. The weight change was 0.04 g, demonstrating to the authors that the TMM4 material would not absorb any MUT.

### 2.2. Biosensor’s Measurement Characteristics

In this work, S-parameter measurements in units of decibels (dB) were taken using a Keysight (Agilent) E5071C ENA, 4-port, ranging from 300 kHz–20 GHz Vector Network Analyzer (VNA). Since dB is a logarithmic unit, the authors converted the S-parameter measurements into a voltage ratio (unitless or V/V), specifically V_2_^+^/V_1_^−^, for easier comparison of results on a linear scale. 

The resulting measured voltages, V_2_^+^ and V_1_^−^, reflect the resulting E- and H-field changes occurring during transmission and, ultimately, whether the MUTs affected these voltages during exposure. For example, if an increase in voltage is measured, it is concluded that the E-field also increased in strength. Alternatively, if the current traveling down the microstrip transmission line increased, it would be concluded that the strength of the magnetic field also increased. An increase in electric or magnetic field strength indicates that the field lines have greater density and will carry more charge. In the case of the proposed RF biosensor, an increase in E-field, for example, would increase the electric charge passing through the MUT within the microliter well.

The proposed RF biosensor has one transmitting and one receiving port, making it a 2-port network. Solving an N-port matrix specifically for a 2-port system,

(1) $$S=\left[\begin{array}{cc} \frac{V_{1}^{-}}{V_{1}^{+}} & \frac{V_{2}^{-}}{V_{1}^{+}}\\ \frac{V_{1}^{-}}{V_{2}^{+}} & \frac{V_{2}^{-}}{V_{2}^{+}} \end{array}\right]$$

gives information about the S_21_ signal, which is the reflection coefficient. Specifically, this ratio communicates the voltage received at Port 2 respective to the input voltage from Port 1.

The following data manipulation took place to convert the experimentally gathered S_21_ measurements, in dB, into voltage ratio values. First, setting S_21_ measurements (in dB) equal to an equivalent statement

(2) $$S_{21}(\mathrm{dB})=20\log\frac{V_{2}^{+}}{V_{1}^{-}}$$

shows the desired voltage ratio will be in terms of the fraction $\frac{V_{2}^{+}}{V_{1}^{-}}$. Solving to isolate this voltage ratio fraction into terms that can be inputted by the S_21_ (dB) measurements gives the following:

(3) $$\frac{V_{2}^{+}}{V_{1}^{-}}=10^{\frac{\mathrm{dB}}{20}}$$

### 2.3. Characteristics as Told by Voltage Ratios

The voltage ratio values will describe how the MUT inside the microliter well behaves during exposure to the broadband sweep. Looking at V_2_^+^ and V_1_^−^ individually gives information about what is occurring at each port. 

If voltage ratio is

(4) $$\left\{\begin{array}{c} \text{>}1,\;\mathrm{says}\;V_{2}^{+}\;\mathrm{is}\;\mathrm{larger}\;\mathrm{than}\;V_{1}^{-}\\ \text{<}1,\;\mathrm{says}\;V_{2}^{+}\;\mathrm{is}\;\mathrm{smaller}\;\mathrm{than}\;V_{1}^{-}\\ =1,\;\mathrm{says}\;V_{2}^{+}\;\mathrm{is}\;\mathrm{equal}\;\mathrm{to}\;V_{1}^{-} \end{array}\right\}$$

Recalling energy in a system can be either (1) absorbed, (2) reflected, (3) transmitted, or (4) stored gives insight into how the MUT interacted with the EM wave. For example, if the voltage ratio is <1, the voltage received at Port 2 was less than that transmitted from Port 1, which most likely was caused by absorption or storage of the energy within the MUT and/or dissipated as heat before arriving at Port 2. Whereas, if the voltage ratio is >1, the EM energy transmitted from Port 1 was less than that received at Port 2. The cause of this may be an increase in EM energy during transmission. Finally, if the voltage ratio is equal to 1, the voltage transmitted from Port 1 is equal to the voltage received at Port 2. This indicates that the EM wave did not interact with the MUT and was transmitted from Port 1 to Port 2.

### 2.4. Experimental Design and Setup

A 4-stage verification experimental design protocol took place to ensure the successful verification of the new RF biosensor and is outlined below.

Stage 1: *Full 2-Port Thru Calibration*

Before beginning any experiment, the Keysight Full 2-Port Thru Calibration was performed using Keysight’s 85052D 3.5 mm Calibration Kit and calibration manual. The VNA settings included:

- Frequency Sweep 1.0–17.0 GHz;
- Collect 1601 data points;
- Collect S_11_ and S_21_ magnitude (dB) and phase (degrees).

Stage 1 was repeated before every subsequent stage of the experimentation.

Stage 2: *Repeatability of Successive Measurements*

Four sets of 10 sweeps (40 sweeps total) were taken to establish the repeatability of measuring a response signal after re-pipetting a MUT, specifically an aqueous solution, into the biosensor’s well. MUTs loaded into the microliter well were:

- Deionized water (at ~21 °C);
- Tris-EDTA;
- NaCl diluted in Tris-EDTA at concentrations 0.2 M, 0.6 M, and 1.0 M;
- Leaving the well empty (air-filled).

Stage 3: *Sensitivity of Measurements*

Steps were taken to show the sensitivity of the VNA to measure and save frequency sweeps. Sets of individual runs were taken and compiled to determine the level of sensitivity and the deviation between sweeps for the following MUTs:

- Deionized water (at ~21 °C);
- Tris-EDTA;
- Lambda DNA (no dilution);
- Lambda DNA diluted in Tris-EDTA at dilutions 1:2, 1:6, and 1:10;
- Leaving the well empty (air-filled).

Stage 4: *Reproducing Measurements on Varying Solutions*

Five sets of 40 sweeps each (200 sweeps total) were collected using the same MUTs listed in Stage 3, emphasizing the measurement of increasing concentrations of lambda DNA diluted in Tris-EDTA buffer. This aimed to verify the RF biosensor’s ability to distinguish individual concentrations of a MUT. The data from this experiment was also used to calculate the signal contributed by the lambda DNA alone by removing any signal from the Tris-EDTA.

### 2.5. Experimental Setup

**Figure 6:**
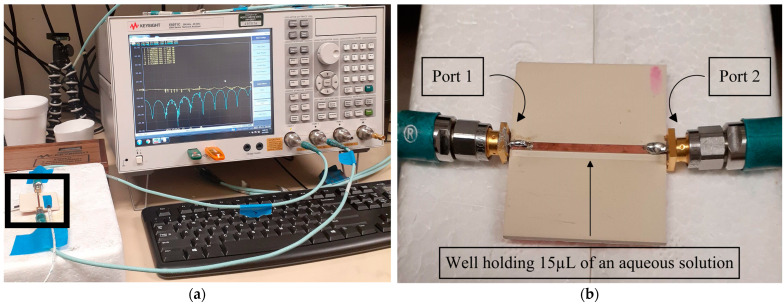
(**a**) The experimental setup consisted of the RF biosensor (left) connected to Port 1 and Port 2 of an E5071C VNA via two 50 Ω coaxial cables. The magnitude and extended phase of S_11_ and S_21_ parameters were saved via as.csv files. (**b**) A close-up of the RF biosensor (outlined by a black box in (**a**)) was connected to the VNA’s Port 1 and 2 with coaxial cables.

The main portion of the experimental setup consisted of the RF biosensor connected to the E5107C Vector Network Analyzer (4-port, 100 kHz–20 GHz) with two 3.5 mm 50 Ω coaxial cables, as shown in Figure 6. Other materials used in experimental testing were a Keysight 85052D 3.5 mm Calibration Kit, a P200 Micropipette and P200 micropipette tips (20–200 µL), deionized water from a Q-POD Ultrapure Water Remote Dispenser (18.2 MΩ/cm @ 25 °C), Tris-EDTA Buffer, New England Biolabs lambda DNA containing 1250 µg (Product # N3011L), iodized sodium chloride, Kimtech wipes, 70% Ethanol spray, and a digital 2-Channel K-Type Thermocouple (58–2372 °F).

## 3. Results and Analysis

### Repeatability and Sensitivity Measurements

To verify a measurable difference between a loaded versus unloaded well, an empty (or air-filled) well measurement set was collected. Next, a matrix of S_21_ measurements holding 40 sweeps of 1601 values (a 40 × 1601 matrix), which was converted from dB into the voltage ratio ($\frac{V_{2}^{+}}{V_{1}^{-}}$), was collected for each aqueous solution tested. A parametric analysis was performed on each dataset, including calculations of a one-sample t-test, a point-by-point mean, standard deviation, and standard error bars.

**Figure 7:**
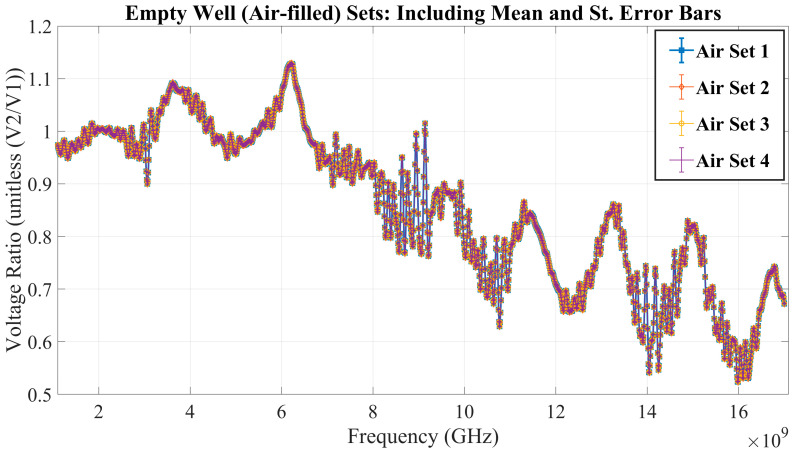
The voltage ratio measurements of the well left empty (air-filled) are shown above. These results show the mean of 10 sweeps per set with 4 sets collected. Standard error bars were attached to each mean to calculate the variability between measurements within a set.

**Figure 8:**
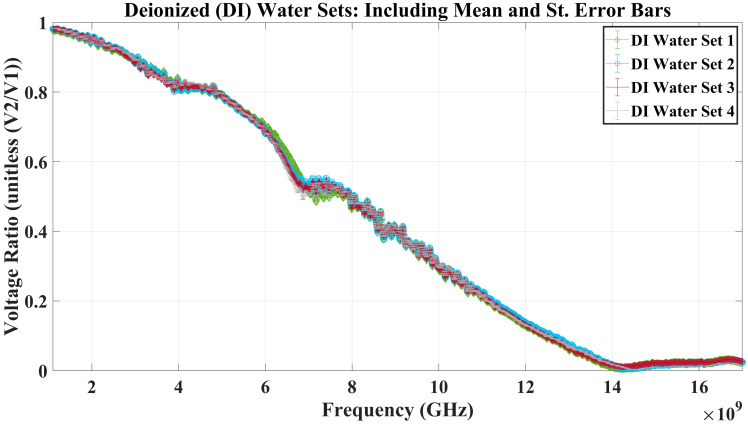
The biosensor was loaded with MUTs, in this case, deionized water, at room temperature (~21 °C). Four sets of ten sweeps were analyzed to determine the repeatability of loading, drying, and measuring the solution under test, along with the mean and standard error bars.

First, a brief inspection of Figure 7 and Figure 8 reveals the RF biosensor’s ability to detect a loaded vs. non-loaded well. The figures show the point-by-point mean and standard error bars for the air-filled well dataset and a deionized (DI) water dataset to determine the repeatability of measurements with MUT replacement. With an almost complete overlap of the standard error envelopes of the four sets from the test cases of the empty well and the deionized water-filled well, it can be concluded that even with MUT replacement, experimental measurement was highly repeatable.

In addition, the analysis included sensitivity calculations based on the experimental measurements gathered from the empty well and DI water-filled well test cases. *Sensitivity* within measurements is the variation between two repeated collections of the same data. Therefore, sensitivity was investigated based on calculations of three types of errors. The absolute average error, standard error range, and relative standard error range—calculated and shown in Table 2—demonstrate the high level of sensitivity seen in the experimental measurements.

**Figure 9:**
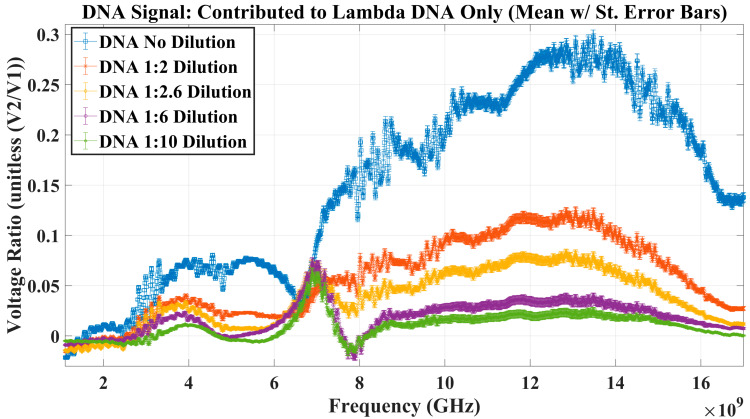
Various MUTs were loaded into the microliter well to determine interaction during EM energy exposure. The experimental data shows distinct voltage ratio trends, demonstrating the RF biosensor’s ability to capture reproducible data.

The final step in verifying the RF biosensor was to ensure the validity of the experimental setup. To complete this, Keysight’s E5071C VNA was (1) calibrated per the procedure in Stage 1 and then (2) connected and loaded with various aqueous solutions, including deionized (DI) water, Tris-EDTA buffer, lambda DNA (not diluted), and lambda DNA diluted in Tris-EDTA buffer in various concentrations. In this portion of the experiment, 5 sets of 40 sweeps were collected, resulting in 200 sweeps. The mean and standard error were calculated for all aqueous solutions loaded into the RF biosensor. The resulting experimentally gathered voltage ratio measurements for the aqueous materials tested (listed in Stages 3 and 4) are seen in Figure 9.

The proposed RF biosensor measured the set of increasing concentrations of lambda DNA diluted in Tris-EDTA buffer with distinction, even with the incorporation of +/− standard error bars. Based on this result, the authors feel the proposed RF biosensor meets the criteria of capably collecting repeatable, sensitive, and reproducible data measurements.

**Figure 10:**
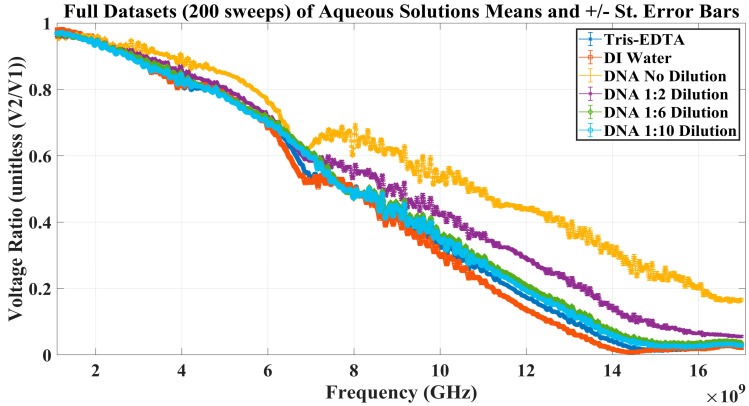
The RF biosensor demonstrates enough measurement sensitivity to distinguish varying concentrations of lambda DNA contained in the well. Frequencies of interest are also seen, showing increased interaction between the MUTs and RF energy.

A secondary interest of this paper was to determine whether increased interaction levels were observed between an aqueous solution, specifically aqueous lambda DNA, at specific frequencies occurring within the RF broadband sweep. The signal mean of the lambda DNA diluted in the Tris-EDTA dataset was subtracted from the mean of the Tris-EDTA buffer dataset to calculate the estimated DNA response signal. Since the lambda DNA was manufactured and shipped in a dehydrated state and was reconstituted in Tris-EDTA, the authors infer that this difference of means is the signal suggesting the behavior of the lambda DNA alone. After the above-mentioned algebraic manipulation, to remove the influence of the diluent, Tris-EDTA, the resulting interaction signal contributed by lambda DNA alone was plotted in Figure 10.

These results show that this biosensor can measure interactions of electromagnetic energy and MUTs in microliter quantities with high repeatability and sensitivity. The innovation value is that one needs to use only microliter samples for testing.

## 4. Discussion

During the RF biosensor repeatability verification stage, Stage 2, a noticeable difference in voltage measurements was observed when comparing the empty well (filled with air) versus the microliter well filled with deionized (DI) water. This difference demonstrated the biosensor’s ability to distinguish the difference between whether the well was loaded with an aqueous solution or not. Also calculated during this stage was the low relative standard error range observed between sets, even though the aqueous solution in the well was replaced between each set, introducing a possible source of error. Overall, this demonstrated that the experimental protocol could capture highly repeatable data throughout lab testing. Similarly, maximum standard error values calculated on the scale of 10^−4^ to 10^−5^ show high sensitivity in the collection of measurements, satisfying Stage 3 of experimentation. The gathered datasets and error calculations give the authors confidence in the RF biosensor’s ability to collect repeatable and sensitive measurements.

In Stage 4, the reproducibility of the gathered measurements was investigated. Of the MUTs measured, varying concentrations of aqueous lambda DNA diluted in Tris-EDTA, as seen in Figure 9, were distinguishable. The E5071C VNA collected signals with small standard errors and a noticeable distinction between the various aqueous MUTs loaded into the microliter well. 

The lambda DNA signal was approximated to determine whether the RF biosensor could detect interaction levels of lambda DNA diluted in Tris-EDTA of various concentrations. This was done by calculating the mean and standard error for each aqueous solution that was tested in the well. Next, since each lambda DNA solution was resuspended in the Tris-EDTA buffer, the dataset mean of each lambda DNA concentration was subtracted from the mean of the Tris-EDTA buffer only. The resulting waveforms, shown in Figure 10, display the effect of interaction from DNA alone, from each dilution, as a signal. Detectable differences were seen as the MUT changed in the concentration of lambda DNA loaded in the microliter well. The highest concentrations of aqueous lambda DNA resulted in larger voltage ratio measurements and lower concentrations in smaller voltage ratio values.

Finally, the results suggest that the interaction between radio frequencies and lambda DNA changes with respect to the frequency transmitted through the aqueous MUT. If the voltage ratio ($\frac{V_{2}^{+}}{V_{1}^{-}}$) measured in the above experimentation had a value less than one, it was understood that the voltage at Port 2 ($V_{2}^{+}$) is smaller than the voltage at Port 1 ($V_{1}^{-}$). This indicated to the authors that absorption or storage may be occurring within the system; however, it is not possible to concretely conclude with the experimental data collected. Therefore, in Stage 4, since lambda DNA was suspended in Tris-EDTA, the MUT was tested in an aqueous form—meaning each element of the solution may contribute to the interaction with RF energy in a unique way. This suggests, due to the structure and polarity of Tris-EDTA and lambda DNA, all voltage ratio values greater than zero (shown in Figure 9) indicated radio frequencies where the lambda DNA part of the solution was interacting in a greater way than the Tris-EDTA buffer. Conversely, when the voltage ratio values became less than zero, the Tris-EDTA buffer interacted with those specific radio frequencies more than the lambda DNA. 

## 5. Conclusions

Based on the experimentation results, the measured data for the repeatability, sensitivity, and reproducibility studies gave strong evidence to verify the functionality of the RF biosensor. Therefore, it is concluded that the proposed RF biosensor is valid and offers a method of broadband electrical impedance/dielectric testing not available before. Further design development will incorporate features for integration into a portable testing/measurement system.

This biosensor collected data from various liquids, with the ability to distinguish between different concentrations of the same MUT. Also, the experimental protocol for collecting data with the RF biosensor resulted in highly repeatable and sensitive data. In the future, more MUTs will be tested, including oils, proteins, and other aqueous solutions, to test the RF biosensor’s ability to test a wide variety and diversity of liquids.

After converting to voltage ratio signals, the response from lambda DNA alone interacting with RF energy was plotted. This showed that as the concentration of lambda DNA in the aqueous solution increased, the overall voltage ratio signal also increased, which suggests specific frequencies of interest where lambda DNA increasingly interacts with radio frequencies. Now that these frequencies of interest have been noted, future experimentation is of great interest to determine whether the mechanism causing increased interaction levels at these frequencies can be identified.

## Figures and Tables

**Table 1:** Microstrip Transmission Line RF Biosensor Dimensions.

Symbol	Quantity	Simulation Dimensions/Measurements
L1	Trace length	40.0 mm
W1	Trace width	1.7965 mm
D1	Trace thickness	17.5 µm
L2 × W2 × D2	Well dimensions	39.0 × 1.5 × 0.25 mm
D3	Substrate height	1.0 mm
εr	Substrate dielectric (Rogers TMM4)	4.7 (unitless)
Z	Impedance	49.9 Ω
v	Well volume	15 µL

**Table 2:** Calculations of Absolute Average, Standard, and Relative Standard Error.

Error Type	Air-Filled Well	DI Water
Absolute Average Error	5.08 × 10^−5^	2.75 × 10^−4^
Min/Max Standard Error Range	6.54 × 10^−6^ to 1.85 × 10^−4^	1.25 × 10^−5^ to 7.40 × 10^−4^
Relative Standard Error Range (%)	0.00065% to 0.025%	0.0013% to 0.36%

## Data Availability

Not applicable.

## References

[1] Mehrotra P., Chatterjee B., Sen S. (2019). EM-Wave Biosensors: A Review of RF, Microwave, mm-Wave and Optical Sensing. Sensors.

[2] Kupfer K. (2005). Electromagnetic Aquametry: Electromagnetic Wave Interaction with Water and Moist Substances.

[3] Ebrahimi A., Withayachumnankul W., Al-Sarawi S., Abbott D. (2014). High-Sensitivity Metamaterial-Inspired Sensor for Microfluidic Dielectric Characterization. IEEE Sens. J..

[4] Withayachumnankul W., Jaruwongrungsee K., Tuantranont A., Fumeaux C., Abbott D. (2013). Metamaterial-based microfluidic sensor for dielectric characterization. Sens. Actuators A Phys..

[5] Subbaraj S., Ramalingam V.S., Kanagasabai M., Sundarsingh E.F., Selvam Y.P., Kingsley S. (2016). Electromagnetic Nondestructive Material Characterization of Dielectrics Using EBG Based Planar Transmission Line Sensor. IEEE Sens. J..

[6] Guha S., Jamal F.I., Wenger C. (2017). A Review on Passive and Integrated Near-Field Microwave Biosensors. Biosensors.

[7] Konoplev G., Agafonova D., Bakhchova L., Mukhin N., Kurachkina M., Schmidt M.-P., Verlov N., Sidorov A., Oseev A., Stepanova O. (2022). Label-Free Physical Techniques and Methodologies for Proteins Detection in Microfluidic Biosensor Structures. Biomedicines.

[8] Alahnomi R.A., Zakaria Z., Yussof Z.M., Althuwayb A.A., Alhegazi A., Alsariera H., Rahman N.A. (2021). Review of Recent Microwave Planar Resonator-Based Sensors: Techniques of Complex Permittivity Extraction, Applications, Open Challenges and Future Research Directions. Sensors.

[9] Salim A., Lim S. (2018). Review of Recent Metamaterial Microfluidic Sensors. Sensors.

[10] Muñoz-Enano J., Vélez P., Gil M., Martín F. (2020). Planar Microwave Resonant Sensors: A Review and Recent Developments. Appl. Sci..

[11] Saha S., Cano-Garcia H., Sotiriou I., Lipscombe O., Gouzouasis I., Koutsoupidou M., Palikaras G., Mackenzie R., Reeve T., Kosmas P. (2017). A glucose sensing system based on transmission measurements at millimetre waves using micro strip patch antennas. Sci. Rep..

[12] Freese J., Jakoby R., Blocher H.-L., Wenger J. (2000). Synthesis of microstrip series-fed patch arrays for 77 GHz-sensor applications. Proceedings of the 2000 Asia-Pacific Microwave Conference, Proceedings (Cat. No.00TH8522).

[13] Zarifi M.H., Farsinezhad S., Shankar K., Daneshmand M. (2015). Liquid Sensing Using Active Feedback Assisted Planar Microwave Resonator. IEEE Microw. Wirel. Compon. Lett..

[14] Zhao W.-S., Gan H.-Y., He L., Liu Q., Wang D.-W., Xu K., Chen S., Dong L., Wang G. (2020). Microwave Planar Sensors for Fully Characterizing Magneto-Dielectric Materials. IEEE Access.

[15] Wadhwani K., Hussaini S., Mazumder A., Syed A. (2022). Solvent-Based Optimization of CSRR and IDC RF Bio-Sensors. IEEE Sens. J..

[16] Lee H.-J., Lee J.-H., Moon H.-S., Jang I.-S., Choi J.-S., Yook J.-G., Jung H.-I. (2012). A planar split-ring resonator-based microwave biosensor for label-free detection of biomolecules. Sens. Actuators B Chem..

[17] Torun H., Cagri Top F., Dundar G., Yalcinkaya A.D. (2014). An antenna-coupled split-ring resonator for biosensing. J. Appl. Phys..

[18] Lee H.-J., Lee H.-S., Yoo K.-H., Yook J.-G. (2010). DNA sensing using split-ring resonator alone at microwave regime. J. Appl. Phys..

[19] Kandwal A., Igbe T., Li J., Liu Y., Li S., Liu L.W.Y., Nie Z. (2020). Highly Sensitive Closed Loop Enclosed Split Ring Biosensor with High Field Confinement for Aqueous and Blood-Glucose Measurements. Sci. Rep..

[20] Abduljabar A.A., Rowe D.J., Porch A., Barrow D.A. (2014). Novel Microwave Microfluidic Sensor Using a Microstrip Split-Ring Resonator. IEEE Trans. Microw. Theory Tech..

[21] Mohammadi S., Nadaraja A.V., Luckasavitch K., Jain M.C., Roberts D.J., Zarifi M.H. (2020). A Label-Free, Non-Intrusive, and Rapid Monitoring of Bacterial Growth on Solid Medium Using Microwave Biosensor. IEEE Trans. Biomed. Circuits Syst..

[22] Wei Z., Huang J., Li J., Xu G., Ju Z., Liu X., Ni X. (2018). A High-Sensitivity Microfluidic Sensor Based on a Substrate Integrated Waveguide Re-Entrant Cavity for Complex Permittivity Measurement of Liquids. Sensors.

[23] Silavwe E., Somjit N., Robertson I.D. (2016). A Microfluidic-Integrated SIW Lab-on-Substrate Sensor for Microliter Liquid Characterization. IEEE Sens. J..

[24] Kobel S., Lutolf M.P. (2011). Biomaterials meet microfluidics: Building the next generation of artificial niches. Curr. Opin. Biotechnol..

[25] Bakir M. (2017). Electromagnetic-Based Microfluidic Sensor Applications. J. Electrochem. Soc..

[26] Grenier K., Dubuc D., Poleni P.-E., Kumemura M., Toshiyoshi H., Fujii T., Fujita H. (2009). Integrated Broadband Microwave and Microfluidic Sensor Dedicated to Bioengineering. IEEE Trans. Microw. Theory Tech..

[27] Wu J., Gu M. (2011). Microfluidic sensing: State of the art fabrication and detection techniques. J. Biomed. Opt..

[28] Zarifi M.H., Sadabadi H., Hejazi S.H., Daneshmand M., Sanati-Nezhad A. (2018). Noncontact and Nonintrusive Microwave-Microfluidic Flow Sensor for Energy and Biomedical Engineering. Sci. Rep..

[29] Ebrahimi A., Scott J., Ghorbani K. (2019). Ultrahigh-Sensitivity Microwave Sensor for Microfluidic Complex Permittivity Measurement. IEEE Trans. Microw. Theory Tech..

[30] Chretiennot T., Dubuc D., Grenier K. (2013). A Microwave and Microfluidic Planar Resonator for Efficient and Accurate Complex Permittivity Characterization of Aqueous Solutions. IEEE Trans. Microw. Theory Tech..

[31] Jang C., Lee H.-J., Yook J.-G. (2021). Radio-Frequency Biosensors for Real-Time and Continuous Glucose Detection. Sensors.

[32] Li Y., Yao Z., Yue W., Zhang C., Gao S., Wang C. (2020). Reusable, Non-Invasive, and Ultrafast Radio Frequency Biosensor Based on Optimized Integrated Passive Device Fabrication Process for Quantitative Detection of Glucose Levels. Sensors.

[33] Pearson M., Ewert D., Striker R., Braaten B. (2021). Development of A RF Biosensor Design to Detect Changes in Scattering Parameters of Aqueous Materials During Radio Frequency Wave Exposure. Biomed. Sci. Instrum..

[34] Balanis C.A. (2012). Advanced Engineering Electromagnetics.

[35] Mechanical and Laser Machining in One System LPKF ProtoMat D104. https://www.lpkf.com/fileadmin/mediafiles/user_upload/products/pdf/DQ/flyer_lpkf_protomat_d104_en.pdf.

[36] Rogers TMM Data Sheet.pdf. https://www.rogerscorp.com/advanced-electronics-solutions/tmm-laminates/tmm-4-laminates.

